# Gestational age, intrauterine growth and body composition at 11 years of age

**DOI:** 10.11606/s1518-8787.2022056004022

**Published:** 2022-11-18

**Authors:** Caroline Cardozo Bortolotto, Iná da Silva dos Santos, Juliana dos Santos Vaz, Alicia Matijasevich

**Affiliations:** I Universidade Federal de Pelotas Programa de Pós-Graduação em Epidemiologia Departamento de Medicina Social Pelotas RS Brasil Universidade Federal de Pelotas. Programa de Pós-Graduação em Epidemiologia. Departamento de Medicina Social. Pelotas, RS, Brasil; II Universidade Federal de Pelotas Faculdade de Nutrição Departamento de Nutrição Pelotas RS Brasil Universidade Federal de Pelotas. Faculdade de Nutrição. Departamento de Nutrição. Pelotas, RS, Brasil; III Universidade de São Paulo Faculdade de Medicina Departamento de Medicina Preventiva Pelotas RS Brasil Universidade de São Paulo. Faculdade de Medicina. Departamento de Medicina Preventiva. Pelotas, RS, Brasil

**Keywords:** Child Development, Body Composition, Gestational Age, Plethysmography, Cohort Studies

## Abstract

**OBJETIVE:**

To assess the association of gestational age (GA) and intrauterine growth with body composition at 11 years of age.

**METHOD:**

Analysis of data from the 2004 Pelotas birth cohort, whose outcomes were fat mass (FM, kg), fat mass index (FMI, kg/m^2^), fat-free mass (FFM, kg), fat-free mass index (FFMI, kg/m^2^) – measured by air displacement plethysmography – and body mass index for age (BMI/age, Z-score). The exposures of interest were the gestational index (GA) of infants born at less than 33 weeks, from 34 to 36 and from 37 to 41, and intrauterine growth categorized as small (SGA), adequate (AGA) and large (LGA) for gestational age. Analysis of variance was used to compare means and linear regression was used to assess the strength of association. The analyses were adjusted according to variables collected at birth, such as monthly family income, maternal characteristics – education, age, pre-gestational body mass index (BMI), weight gain during pregnancy, smoking during pregnancy, type of delivery, and parity – and adolescent characteristics – skin color and birth weight. For analysis, FM and FMI underwent logarithmic transformation due to data asymmetry.

**RESULTS:**

A total of 3,401 adolescents were analyzed, including boys and girls born at less than 33 weeks, with lower FM and FFM means than those born at term. However, in the adjusted analyses, there was no association between GA and any of the outcomes in either sex. LGA boys had a 10.5% higher FMI (p = 0.026) and +0.3 BMI/age Z-score (p = 0.019) as compared to AGA boys, and LGA girls had +0.3 kg/m ^2^ of FFMI (p = 0.039) than AGA girls.

**CONCLUSION:**

GA was not associated with body composition at 11 years of age. However, LGA boys had higher BMI and BMI/age Z-score, and LGA girls had higher FFMI than AGA girls.

## INTRODUCTION

The prevalence of overweight in young people has increased in recent decades. In 2016, it was estimated that 340 million children and adolescents aged between 5 and 19 years, worldwide, were overweight or obese^1^. In Brazil, national surveys have shown, in the last three decades, an increase in the incidence of overweight in children and adolescents between 6 and 19 years, regardless of the macro-region or family income^2^.

According to the “Theory of the fetal origin of diseases*” (*Developmental origins of Health and Disease – DoHaD), excess weight and body composition in early life are associated with cardiovascular and metabolic diseases in adulthood^3^. It is between late childhood and early adolescence that there is a great variation in body composition^4^, a period considered critical for the beginning of the development of obesity in both sexes^5^, although the distribution of body fat occurs differently across boys and girls^4^. In this, in addition to factors related to lifestyle, such as eating habits, physical activity and sedentary behavior, the role of biological factors, such as gestational age (GA), in the occurrence of overweight in childhood and adolescence, has also been investigated^6-8^.

Longitudinal^7,8^and cross-sectional^6,9^studies show that the association between GA and body composition, assessed by indirect methods – air displacement plethysmography^7^and X-ray dual absorption densitometry (Dexa) –^6,8,9^, is still not well established. While some studies have detected lower body fat and fat-free mass (FFM) measurements in preterm infants – compared to full-term infants^6,7^–, others have not found an association both in early adolescence, at 11 years^9^, and at the end of it, at the age of 18^8^.

In addition, the relationship between GA, intrauterine growth and body composition in adolescence is rarely found in the literature. Thus, the aim of this study was to investigate the association of GA and intrauterine growth with body composition in 11-year-old boys and girls in the 2004 Pelotas birth cohort.

## METHODS

### Sample Characteristics

The research was developed based on the analysis of data from the birth cohort of Pelotas in 2004, a city with 328,275 inhabitants and located in south Brazil^10^. That year, a birth cohort was started that included all newborns from hospital deliveries, which took place between January 1 and December 31, and which followed mothers who lived in the urban area of the municipality and in the Jardim América neighborhood, adjacent to Pelotas. but belonging to the neighboring municipality of Capão do Leão. A total of 4,231 newborns, i.e., 99.2% of all births in the city that year, were included in the study, in addition to standardized interviews conducted with mothers during their hospital stay (perinatal study) to investigate socioeconomic, demographic and behavioral characteristics. The newborns were examined within 24 hours after delivery by the study team, under the supervision of a pediatrician, being followed up at birth, at 3, 12, 24 and 48 months, and at 6 and 11 years of age, with follow-up rates ranging from 87% to 96% – a detailed description of the methodology is provided elsewhere^11^.

For the current study, those followed at 11 years of age and who had sufficient information for at least one of the indices analyzed in this research were eligible; twins and those who had some congenital malformation that could interfere with feeding and walking were excluded. Considering this, at 11 years of age, of the 4,231 members of the cohort, 567 were lost or refused to participate in the follow-up, 84 were twins and 81 did not have data on the essential variables for carrying out the research. These losses occurred, for example, in cases in which the adolescent was unable to stand up to check weight and height, which are essential measures for air displacement plethysmography, or when they were unable to stay inside the air chamber. Adding these cases to the 98 deaths that occurred between birth and 11 years of age, there remained the 3,401 children (80.4% of the total) included in the present analysis.

### Body Composition

At the 11-year follow-up, body composition was assessed using air displacement plethysmography (Bod Pod^®^, Cosmed), performed by specifically trained technicians; a safe, fast and non-invasive method that can be applied in different population groups, such as obese people, children, adults and elderly people^12^. In order to evaluate it, the participants remained inside the device, a closed chamber, for a few seconds, without moving. To then measure with adequate accuracy and minimize disparities in body volume measurement, it was necessary to eliminate the volume effect of clothing, hair, body surface, and lungs. On this account, participants were provided with sets of rubber caps – such as those for swimming –, shorts and spandex t-shirts, made especially for the study. To predict the volume of thoracic gas, a formula was used based on the participants age, sex and height^13^and, to estimate body fat and FFM, the Lohman equation was used^14^.

The outcomes of this study were fat mass (FM) in kg, fat mass index (FMI) in kg/m^2^, FFM in kg, fat-free mass index (FMI) in kg/m^2^ and body mass index (BMI) for age (Z-score). FMI and FFMI were calculated from the ratio, respectively, between FM (kg) and FFM (kg) and height in square meters (m^2^). BMI/age was calculated as the ratio between weight in kg and height in m^2^. And age- and sex-specific BMI/age Z-scores were calculated according to growth curves published by the World Health Organization (WHO) in 2006^15^, using the ANTHRO 2007 software.

Weight was assessed using a high-precision scale (0.01 kg), coupled to the BodPod^®^ equipment; height was measured twice, using a Harpenden metal stadiometer (Holtain, Crymych, United Kingdom) with a maximum height of 2.06 m and an accuracy of 1 mm; finally obtaining the average of these two measurements. In cases where the difference between the two measurements was greater than 1 cm, a third measurement was performed.

### Gestational Age and Intrauterine Growth

The algorithm proposed by the National Center for Health Statistics (NCHS)^16^was used to estimate GA at birth, based on the date of the last menstrual period (LMP) – whenever consistent with weight, length and head circumference at birth, according to the standard curves of these parameters for each week of GA^17^. If the estimate was unknown or inconsistent, clinical maturity was estimated based on the Dubowitz method^18^_._ In this analysis, participants born before 37 weeks of gestation were classified as preterm. Thus, GA was categorized into three groups: those born at less than 33 weeks, which include moderately preterm, very preterm and extremely preterm infants; those at 34 to 36 weeks, late preterm infants; and those at 37 to 41 weeks, term newborns^19^.

Intrauterine growth was defined according to the of INTERGROWTH-21^20^ parameters, which include birth weight according to GA and sex and the classification of participants as small for gestational age (SGA; birth weight less than 10%), adequate for gestational age (AGA; birth weight between 10% and 90%) and large for gestational age (LGA; birth weight greater than 90%). Birth weight was collected using electronic pediatric scales (Harpenden@) with an accuracy of 10g, checked daily by the research team, and those weighing less than 2,500g were considered low birth weight (LBW).

### Potential Confounders

The adjustment variables were defined according to a conceptual model created by the authors, based on the literature^21^. Therefore, family income was obtained by adding the income of people who lived in the same house, in the month before the perinatal interview, and later divided into quintiles (Q1, for families with lower incomes, and Q5, for families with higher incomes)^22^.

Maternal characteristics, obt^a^ined in the perinatal interview, included schooling, collected in complete years with approval and classified into groups from 0 to 4 years, 5 to 8, 9 to 11 and greater than or equal to 12 years; age in complete years, categorized as younger than 20 years old, from 20 to 34 years old, and greater than or equal to 35 years old; smoking, if they smoked at least one cigarette a day, every day, in any trimester of pregnancy; type of delivery, whether normal or cesarean, extracted from hospital records; parity, that is, number of live or dead births, categorized as 0, 1, 2, 3 and greater than or equal to 4; weight at the beginning and end of pregnancy, extracted from the Pregnancy Card, the last record being considered the final weight, or, when not available, the mother’s report; weight gain during pregnancy, calculated as the difference between final and initial weights; pre-pregnancy BMI, classified as underweight (≤ 18.49 kg/m^2^), adequate (18.5–24.9 kg/m^2^), overweight (25.0–29.9 kg/m^2^) or obesity (≥ 30 kg/m^2^)^23^; and maternal height, measured at home by the research team, in follow-ups performed three months after delivery.

Finally, information on the adolescent’s sex was collected directly from the birth medical record; and the color of their skin, self-reported by the mother as white, brown, black or other, information obtained in the follow-ups carried out at the age of six. For girls, in the follow-up of 11 years old, they were asked if they had already had menarche. It is also worth mentioning that, as only 182 mothers reported skin color as “other”, we ended up grouping these adolescents in the “brown” category for analysis purposes.

### Statistical Analysis

Statistical analyses were performed using Stata version 16.0 (Stata Corp., College Station, USA). After describing the follow-up sample at 11 years of age, the means and 95% confidence intervals (95%CI) of FM, FMI, FFM, FFMI and BMI/age Z-score were described, according to GA and intrauterine growth, separated by sex; to compare the means, analysis of variance (Anova) was used.

As 251 girls (15.4%) had already had menarche, analyses were performed to investigate the existence of an interaction between GA and menarche history on outcomes. As there was no statistical evidence of interaction, all girls were included in the analyses, regardless of menarche history. However, there was an interaction (p < 0.20) between GA and sex on the FFMI outcomes (p = 0.145) and BMI/age Z-score (p = 0.092), causing all analyses to be stratified by sex.

The strength of association between the independent variables and the outcomes was verified by linear regression, through beta coefficients (β) and 95%CI; FM and FMI data underwent logarithmic transformation, as they present asymmetric distribution; and the beta coefficients of the raw and adjusted analyses are shown in their exponential form. Thus, the adjusted analyzes were hierarchical, the first level comprising maternal education and age and family income; the second level, pre-gestational BMI, smoking during pregnancy, weight gain during pregnancy, type of delivery and parity; and the third, the adolescent’s skin color and birth weight.

Current height was included in the fourth level, but as it is used in the construction of the FMI, FFMI and BMI, in the analyses for these outcomes, height was not included as a potential confounder. Likewise, when the exposure of interest was intrauterine growth, birth weight was not included as a confounder in the adjusted models. Therefore, at each level, the p-value was verified, removing the variables with the highest p-value, one by one, from the analysis model. However, the variables associated with the outcome with p-value <0.20 were kept in the analysis to control for possible confounding effects. In the course of this, a significance level of 5% was assumed in the statistical analyses.

### Ethical Aspects

All follow-ups of the 2004 Pelotas birth cohort were approved by the Research Ethics Committee of the Faculty of Medicine of the Federal University of Pelotas, affiliated with the National Research Ethics Commission (Conep). Therefore, the children’s parents or legal guardians gave written consent at each follow-up and consent was obtained, also in writing, from participants aged at least 11 years (letter number 889,753).

## RESULTS

In the analyzed sample (n = 3,401), 51.7% (n = 1,757) of the participants were boys, with about 45% (n = 1,523) born by cesarean section, 13.5% (n = 430) preterm, and 8.5% (n = 280), SGA (Table 1). Regarding maternal variables, most mothers had between 5 and 8 years of schooling (41.2%) and were aged between 20 and 34 years (67%). About a quarter of them (25.3%) were overweight and 11.5% were obese. In addition, the prevalence of maternal smoking during pregnancy was 26.9%, and mothers gained an average of 10.5 kg during pregnancy (SD = 5.6).

**Table 1:** Characteristics of mothers and participants in the 2004 Pelotas birth cohort, at birth, followed up at 11 years of age.

Features	Included in the analysis n = 3.401	
Maternal education (completed years)^a^	(n = 3,371)	%
0–4	497	14.7
5–8	1,387	41.2
9–11	1,162	34.5
≥ 12	325	9.6
Maternal age (years)	(n = 3,399)	
< 20	648	19.1
20–34	2,279	67.0
≥ 35	472	13.9
Pre-gestational BMI	(n = 2,407)	
Low weight (≤ 18.49 kg/m^2^)	91	3.8
Adequate (18.5–24.9 kg/m^2^)	1,432	59.4
Overweight (25.0–29.9 kg/m^2^)	608	25.3
Obesity (≥ 30 kg/m^2^)	276	11.5
Smoking in pregnancy	(n = 1,757)	
No	2,487	73.1
Yes	914	26.9
Type of delivery	(n = 1,878)	
Normal	1,878	55.2
Caesarean	1,523	44.8
Parity (number of children born alive or dead)	(n = 3,400)	
1	1,345	39.6
2	919	27.0
3	539	15.9
≥ 4	597	17.6
Weight gain during pregnancy (kg)	(n = 3,310)	
(mean, SD)	10.5 (5.6)	-
Family income (quintiles)	(n = 3,401)	
Q1 (lower income)	657	19.3
Q2	682	20.1
Q3	680	20.0
Q4	730	21.5
Q5 (higher income)	652	19.2
Skin color	(n = 3,384)	
White	2,287	67.6
Brown	677	20.0
Black	420	12.4
Birth weight (g)	(n = 3,401)	
< 2,500 (low weight)	260	7.6
2,500–2,999	832	24.5
3,000–3,499	1,369	40.3
3,500–3,999	768	22.6
≥ 4,000	172	5.1
GA (weeks)	(n = 3,186)	
≤ 33	71	2.2
34–36	359	11.3
37–41	2,756	86.5
Intrauterine growth	(n = 3,294)	
SGA	280	8.5
AGA	2,446	74.3
LGA	568	17.2

SD: standard deviation; BMI: body mass index; GA: gestational age; SGA: small for GA; AGA: adequate for gestational age; LGA: large for gestational age.

Table 2 shows the FM and FMI medians and BMI/age Z-score means, with the respective 95%CI, according to the exposures of interest and separately for boys and girls. As for GA, the lowest means and medians were observed among preterm infants compared to full-term infants. Boys born at or less than 33 weeks had smaller FM and FMI medians (6.1 kg and 3.3 kg/m^2^, respectively) than those born at term (p < 0.001 and p < 0.001). In girls, only FM was different between the GA categories, with the medians of those born at or less than 33 and at 34 to 36 weeks being 7.6 kg and 9.2 kg smaller, also respectively, compared to those born at term (p = 0.024). The BMI/age Z-score was statistically different only among boys, and it was 0.3 point lower among those born at or less than 33 weeks than in those born at term (p < 0.001).

**Table 2:** Measures of central tendency of FM, FMI and BMI for age (Z-score) at 11 years, according to sex, gestational age and intrauterine growth, in the 2004 Pelotas birth cohort.

Variable	Boys				Girls			
	
	Total	FM (kg)	FMI (kg/m^2^)	BMI/A (Z-score)	Total	FM (kg)	FMI (kg/m^2^)	BMI/A (Z-score)
							
	n	Median (95%CI)	Median (95%CI)	Median (95%CI)	n	Median (95%CI)	Median (95%CI)	Median (95%CI)
**GA**		**9.6 (3.0 to 26.5)**	**4.5 (1.6 to 11.5)**	**0.8 (0.7 to 0.9)**		**10.0 (3.8 to 27.1)**	**4.6 (1.9**^**a**^ **11.9)**	**0.7 (0.6 to 0.7)**
**(Weeks)**		**p < 0.001**^**a**^	**p < 0.001**^**a**^	**p < 0.001**^**b**^		**p = 0.024**^**a**^	**p = 0.082**^**a**^	**p = 0.167**^**b**^
≤ 33	38	6.1 (1.9 to 40.3)	3.3 (1.0 to 14.9)	0.3 (- 0.3 to 0.8)	33	7.6 (3.8 to 20.1)	4.0 (1.9 to 9.4)	0.6 (0.2 to 1.0)
34–36	187	7.6 (2.8 to 24.4)	3.7 (1.5 to 10.4)	0.4 (0.2 to 0.7)	172	9.2 (3.0 to 24.8)	4.3 (1.4 to 10.4)	0.5 (0.3 to 0.7)
37–41	1.425	10.1 (3.1 to 26.5)	4.7 (1.6 to 11.5)	0.9 (0.8 to 1.0)	1.331	10.2 (3.9 to 28.1)	4.7 (1.9 to 12.1)	0.7 (0.6 to 0.8)
**Intrauterine growth**		**p = 0.028**^**a**^	**p = 0.021**^**a**^	**p < 0.001**^**b**^		**p = 0.004**^**a**^	**p = 0.006**^**a**^	**p = 0.001**^**b**^
SGA	142	8.1 (2.9 to 21.9)	3.9 (1.6 to 9.5)	0.5 (0.3 to 0.7)	138	8.9 (2.9 to 20.9)	4.1 (1.5 to 9.1)	0.3 (0.1 to 0.5)
AGA	1.286	9.1 (2.9 to 23.7)	4.4 (1.5 to 10.6)	0.7 (0.6 to 0.8)	1.160	9.7 (3.8 to 24.2)	4.5 (1.9 to 10.7)	0.6 (0.5 to 0.7)
LGA	1.704	10.7 (3.1 to 25.3)	5.0 (1.6 to 11.1)	1.0 (0.8 to 1.2)	292	10.7 (3.8 to 25.4)	4.8 (1.9 to 11.1)	0.7 (0.6 to 0.9)

FM: fat mass; FMI: fat mass index; BMI/A: body mass index for age, in Z-score; GA: gestational age; SGA: small for GA; AGA: adequate for GA; LGA: large for GA; 95%CI: 95% confidence interval.

^a^ Kruskal-Wallis nonparametric heterogeneity test.

^b^ Anova parametric heterogeneity test.

As for intrauterine growth, in both sexes, there were lower mean Z-scores for BMI/age and lower medians for FM and FMI in SGA compared to AGA infants. SGA boys had, on average, 0.3 less BMI/age Z-score and 8.1 kg and 3.9 kg/m^2^ less, respectively, in the FM and FMI medians, compared to AGA boys. Among SGA girls, the mean BMI Z-score was 0.3 point lower and the FM and FMI medians were 8.9 kg and 4.1 kg/m^2^ lower, respectively, than among those born at AGA (Table 2).

As for FFM, the FFM and FMI means were lower in preterm than in full-term boys. On the one hand, boys born at or less than 33 weeks had, on average, 2.2 kg less FFM than those born at term, and the FFM of boys born preterm was 0.4 kg/m^2^lower as compared to term infants. On the other hand, in girls only differences for FFM were observed, with those born at or less than 33 weeks, on average, with 1.2 kg less than those born at term. SGA boys and girls had, respectively, 0.3 kg and 0.6 kg less FFM as compared to AGA infants of the same sex, and the SGA boys’ FFMI was 0.2 kg/m^2^lower than that of term infants (Table 3).

**Table 3:** Measures of central tendency of FFM and FFMI, at 11 years of age, according to sex, gestational age and intrauterine growth, in the 2004 Pelotas birth cohort.

Variable	Boys			Girls		
	
	Total	FFM (kg)	FFMI (kg/m^2^)	Total	FFM (kg)	FFMI (kg/m^2^)
					
	n	Median (95%CI)	Median (95%CI)	n	Median (95%CI)	Median (95%CI)
**GA**		**30.0 (29.8 to 30.2)**	**14.3 (14.2 to 14.3)**		**31.1 (30.8 to 31.4)**	**14.4 (14.3 to 14.4)**
**(Weeks)**		**p < 0.001**^**a**^	**p = 0.001**^**a**^		**p = 0.025**^**a**^	**p = 0.413**^**a**^
≤ 33	38	28.1 (26.0 to 29.5)	13.9 (13.5 to 14.4)	33	30.1 (28.1 to 32.1)	14.6 (13.9 to 15.3)
34–36	187	29.0 (28.3 to 29.7)	13.9 (13.7 to 14.2)	172	30.2 (29.4 to 31.1)	14.2 (14.0 to 14.5)
37–41	1,425	30.3 (30.0 to 30.5)	14.3 (14.2 to 14.4)	1,331	31.3 (30.9 to 31.6)	14.4 (14.3 to 14.5)
**Intrauterine growth**		**p = 0.027**^**a**^	**p = 0.020**^**a**^		**p = 0.018**^**a**^	**p = 0.100**^**a**^
SGA	142	29.4 (28.6 to 30.1)	14.0 (13.8 to 14.2)	138	30.1 (29.1 to 31.1)	14.0 (13.7 to 14.3)
AGA	1,286	29.7 (29.5 to 30.0)	14.2 (14.1 to 14.3)	1,160	30.7 (30.4 to 31.0)	14.3 (14.2 to 14.4)
LGA	1,704	30.6 (30.0 to 31.1)	14.5 (14.3 to 14.6)	292	31.7 (31.0 to 32.4)	14.5 (14.3 to 14.7)

GA: gestational age; FFM: fat-free mass; FFMI: fat-free mass index; SGA: small for GA; AIG: adequate for GA; LGA: large for GA; 95%CI: 95% confidence interval.

^a^ Parametric heterogeneity test: Anova

Tables 4 and 5 show the differences between the outcomes’ means, according to GA and intrauterine growth, in each sex. After adjusting for confounders, there was no association between GA and any of the outcomes, either in boys or girls (Table 4). Regarding intrauterine growth, LGA boys had a 10.5% higher FMI (p = 0.026) and 0.3 more BMI/age Z-score (p = 0.019) than AGA boys. In girls, there was a trend towards a direct increase in FFMI with intrauterine growth, where LGA girls had 0.3 kg/m^2^more than AGA girls (p = 0.039) (Table 5).

**Table 4:** Association between gestational age, body composition and body mass index Z-score for age, at 11 years old, according to sex, in the 2004 Pelotas birth cohort.

GA (Weeks)	FM (kg)^a^		FMI (kg/m^2^)^a^		BMI/A (Z-Score)		FFM (kg)		FFMI (kg/m^2^)	
				
	β (95%CI)		β (95%CI)		β (95%CI)		β (95%CI)		β (95%CI)	
				
	Gross	Adjusted	Gross	Adjusted	Gross	Adjusted	Gross	Adjusted	Gross	Adjusted
**Boys**	**p < 0.001**	**p = 0.119**^**b**^	**p < 0.001**	**p = 0.415**^**c**^	**p < 0.001**	**p = 0.206**^**d**^	**p < 0.001**	**p = 0.134**^**e**^	**p < 0.001**	**p = 0.228**^**f**^
≤ 33	-0.4 (-0.6 to -0.1)	0.0 (-0.3 to 0.1)	-0.3 (-0.5 to -0.1)	0.0 (-0.3 to 0.2)	-0.6 (-1.1 - -0.2)	0.0 (-0.6 to 0.5)	-2.2 (-3.8 -0.7)	-0.7 (-1.3 to -0.1)	-0.4 (-0.8 to 0.1)	-0.3 (-0.6 to 0.0)
34–36	-0.2 (-0.3 to -0.1)	0.0 (-0.2 to 0.0)	-0.1 (-0.2 to 0.0)	0.0 (-0.2 to 0.0)	-0.5 (-0.7 a -0.2)	-0.2 (-0.5 to 0.0)	-1.3 (-2.0 -0.6)	0.0 (-1.3 to 1.3)	-0.4 (-0.6 -0.2)	0.0 (-0.6 to 0.6)
37–41	ref.	ref.	ref.	ref.	ref..	ref.	ref.	ref.	ref.	ref.
**Girls**	**p = 0.022**	**p = 0.983**^**g**^	**p = 0.070**	**p = 0.714**^**h**^	**p = 0.167**	**p = 0.357**^**i**^	**p = 0.025**	**p = 0.574**^**j**^	**p = 0.831**	**p = 0.206**^**k**^
≤ 33	-0.2 (-0.4 to 0.1)	0.0 (-0.3 to 0.3)	-0.1 (-0.3 to 0.1)	0.0 (-0.3 to 0.3)	-0.1 (-0.6 to 0.4)	0.2 (-0.5 to 0.8)	-1.0 (-2.0 -0.1)	0.3 (-0.5 to 1.0)	0.3 (-0.4 to 0.9)	0.2 (-0.2 to 0.6)
34–36	-0.1 (-0.2 to 0.0)	0.0 (0.1 to 0.1)	-0.1 (-0.2 to 0.0)	0.0 (-0.1 to 0.2)	-0.2 (-0.5 to 0.0)	0.1 (-0.2 to 0.4)	-1.2 (-3.3 - 0.9)	0.1 (-1.6 to 1.7)	-0.2 (-0.4; 0.1)	0.3 (-0.5 to 1.2)
37–41	ref.	ref.	ref.	ref.	ref.	ref.	ref.	ref.	ref.	ref.

MG: fat mass; FMI: fat mass index; FFM: fat-free mass; FFMI: fat-free mass index; BMI: body mass index for age, in Z-score; 95%CI: 95% confidence interval; ref.: reference.

Note: Linear trend p-values.

^a^ Logarithmic transformation was performed.

^b^ Adjusted for family income, schooling, age, smoking during pregnancy, weight gain during pregnancy, maternal pre-pregnancy BMI and parity, and adolescent birth weight, skin color, and height.

^c^ Adjusted for family income, education, age, smoking during pregnancy, weight gain during pregnancy, maternal pre-pregnancy BMI and parity, and adolescent birth weight and skin color.

^d^ Adjusted for income, education, smoking during pregnancy, weight gain during pregnancy, maternal pre-pregnancy BMI and parity, and adolescent birth weight.

^e^ Adjusted for schooling, age, smoking during pregnancy, weight gain during pregnancy, maternal pre-pregnancy BMI and parity, and adolescent birth weight, skin color, and height.

^f^ Adjusted for income, education, smoking during pregnancy, weight gain during pregnancy, pre-pregnancy BMI, maternal type of delivery and parity, and adolescent birth weight and skin color.

^g^ Adjusted for schooling, age, smoking during pregnancy, weight gain during pregnancy, maternal pre-pregnancy BMI and parity, and adolescent birth weight and height.

^h^ Adjusted for schooling, age, smoking during pregnancy, weight gain during pregnancy, maternal pre-pregnancy BMI and parity, and adolescent birth weight.

^i^ Adjusted for schooling, smoking during pregnancy, weight gain during pregnancy, maternal pre-pregnancy BMI and parity, and adolescent birth weight.

^j^ Adjusted for education, maternal age, smoking during pregnancy, weight gain during pregnancy, maternal pre-pregnancy BMI and parity, and adolescent birth weight.

^k^ Adjusted for smoking during pregnancy, weight gain during pregnancy, maternal pre-pregnancy BMI and maternal parity, and adolescent birth weight and skin color.

**Table 5:** Association between intrauterine growth, body composition and body mass index Z-score for age, at 11 years old, according to sex , in the 2004 Pelotas birth cohort.

Intrauterine growth	FM (kg)^a^		FMI (kg/m^2^)^a^		BMI/A (Z-score)		FFM (kg)		FFMI (kg/m^2^)	
				
	β (95%CI)		β (95%CI)		β (95%CI)		β (95%CI)		β (95%CI)	
				
	Gross	Adjusted	Gross	Adjusted	Gross	Adjusted	Gross	Adjusted	Gross	Adjusted
**Boys**	**p = 0.003**	**p = 0.083**^**b**^	**p = 0.003**	**p = 0.026**^**c**^	**p < 0.001**	**p = 0.019**^**d**^	**p = 0.027**	**p = 0.356**^**e**^	**p = 0.020**	**p = 0.193**^**f**^
SGA	-0.1 (-0.2 to 0.0)	-0.1 (-0.2 to 0.0)	-0.1 (-0.2 to 0.0)	-0.1 (-0.2 to 0.0)	-0.2 (-0.4 to 0.0)	-0.2 (-0.4 to 0.1)	-0.4 (-1.2 to 0.4)	-0.3 (-0.9 to 0.3)	-0.2 (-0.4 to 0.1)	-0.2 (-0.5 to 0.1)
AGA	ref.	ref.	ref.	ref.	ref.	ref.	ref.	ref.	ref.	ref.
LGA	0.1 (0.0 to 0.2)	0.1 (0.0 to 0.2)	0.1 (0.0 to 0.2)	0.1 (0.1 to 0.2)	0.3 (0.1 to 0.5)	0.3 (0.1 to 0.5)	0.8 (0.2 to 1.4)	0.3 (-0.2 to 0.8)	0.3 (0.1 to 0.5)	0.2 (0.0 to 0.4)
**Girls**	**p = 0.005**	**p = 0.178**^**g**^	**p = 0.006**	**p = 0.067**^**h**^	**p = 0.001**	**p = 0.050**^**i**^	**p = 0.018**	**p = 0.088**^**j**^	**p = 0.100**	**p = 0.039**^**k**^
SGA	-0.1 (-0.2 to 0.0)	-0.1 (-0.2 to 0.0)	-0.1 (-0.2 to 0.0)	-0.1 (-0.2 to 0.0)	-0.3 (-0.5 to 0.0)	0.0 (0.0 to 0.2)	-0.6 (-1.6 to 0.5)	-0.3 (-1.0 to 0.5)	-0.3 (-0.6 to 0.0)	-0.1 (-0.5 to 0.2)
AGA	ref.	ref.	ref.	ref.	ref.	ref.	ref.	ref.	ref.	ref.
LGA	0.0 (0.0 to 0.2)	0.0 (0.0 to 0.2)	0.0 (0.0 to 0.1)	0.1 (0.0 to 0.2)	0.2 (0.0 to 0.3)	0.0 (0.0 to 0.0)	1.1 (0.3 to 1.8)	0.6 (0.0 to 1.1)	0.2 (0.0 to 0.5)	0.3 (0.1 to 0.6)

FM: fat mass; FMI: fat mass index; FFM: fat-free mass; FFMI: fat-free mass index; BMI: body mass index for age, in Z-score; GA: gestational age; SGA: small for GA; AGA: adequate for GA; LGA: large for GA; 95%CI: 95% confidence interval; ref.: reference.

Note: Linear trend p-values.

^a^ Logarithmic transformation was performed.

^b^ Adjusted for family income, education, age, smoking during pregnancy, weight gain during pregnancy, maternal pre-pregnancy BMI and parity, and adolescent’s skin color and height.

^c^ Adjusted for family income, schooling, age, smoking during pregnancy, weight gain during pregnancy, maternal pre-pregnancy BMI and parity, and adolescent skin color.

^d^ Adjusted for family income, schooling, smoking during pregnancy, weight gain during pregnancy, pre-pregnancy BMI and maternal parity.

^e^ Adjusted for family income, schooling, smoking during pregnancy, weight gain during pregnancy, maternal pre-pregnancy BMI and parity, and adolescent birth weight, skin condition, and height.

^f^ Adjusted for family income, schooling, smoking during pregnancy, weight gain during pregnancy, maternal pre-pregnancy BMI and type of delivery, and adolescent birth weight and skin color.

^g^ Adjusted for schooling, age, smoking during pregnancy, weight gain during pregnancy, maternal pre-pregnancy BMI and parity, and adolescent height.

^h^ Adjusted for education, age, smoking during pregnancy, weight gain during pregnancy, pre-pregnancy BMI, maternal type of delivery and parity, and adolescent’s skin color.

^i^ Adjusted for maternal education, smoking during pregnancy, weight gain during pregnancy, pre-pregnancy BMI and parity.

^j^ Adjusted for maternal schooling, age, pre-pregnancy BMI and parity, and adolescent birth weight, skin color, and height.

^k^ Adjusted for smoking during pregnancy, maternal pre-pregnancy BMI and parity, and adolescent birth weight and skin color.

## DISCUSSION

This study analyzed the association of GA and intrauterine growth with body composition in early adolescence, that is, at 11 years of age. After adjusting for confounders, only FMI and BMI/age Z-score were higher among LGA boys compared to AGA ones; and, among girls, only LGA’s FFMI was higher than that of AGA.

The non-association of GA with outcomes is consistent with the findings of other authors^8,9^. In the 1993 Pelotas birth cohort, for example, no association was observed between GA and FM, FFM – measured by air displacement plethysmography – or BMI, in both sexes at the end of adolescence, at 18 years of age^8^. Likewise, a case-control study carried out in Spain found no difference in FM and FFM between preterm and full-term infants aged 7 to 11 years, in the control group assessed by DEXA^9^.

On the other hand, in the same cohort, lower values of FM, IMG, FFM and IMLG, measured by air displacement plethysmography, were found in six-year-old boys born between 34 and 36 weeks, compared to full-term boys^8^. Other studies, with participants aged between 8 and 12 years old, in the United Kingdom^6^, and 11 years old, in England^7^, both carried out by DEXA, also identified lower body fat contents among adolescents born preterm^6,7^.

The plausibility of the association between preterm birth and lower body fat in childhood and adolescence is based on the fact that the storage of energy – fat and glycogen – of nutrients, in addition to the greater deposition of MG and FFM^24^, occur mainly in the last trimester of pregnancy. Thus, termination of pregnancy before 37 weeks would lead to a low reserve of energy and nutrients in preterm infants^3^.

The mechanisms responsible for the difference in the body composition of children born preterm are likely to be multifactorial, such as factors that influence the handling or availability of nutrients, concomitant illnesses such as infection and lung disease, and hormonal influences, for example, the use of post-natal corticosteroids^24^. Therefore, as suggested by Fewtrell et al.^6^, it is possible that the premature interruption of intrauterine fat deposition in preterm infants has a long-term effect on subsequent body fat gain.

Although evidence on the association between GA and body composition in adolescence is still scarce, studies show lower FM among preterm infants in childhood^6,8^and an inversion of the effect of GA on FM in adulthood^8^, so that preterm infants have more FM than full-term infants as adults. This reversal of the effect throughout life may be related to mediating mechanisms, such as education level, alcohol consumption, physical activity and eating habits^25^.

The interactions between GA and sex for the FFMI and BMI/age Z-score, observed in our study, possibly result from the fact that sexual maturation occurs differently across boys and girls^4^. Pubertal phenomena show variability in terms of age at onset and termination and the speed and magnitude with which they are expressed, which can influence anthropometric and body composition changes that differ in the process of growth and development in adolescence^4^. During this period, in both sexes, there is a significant increase in FM, but in boys, it is slower, being overcome by the gain in FFM^4^.

Regarding intrauterine growth, being born LGA is associated with childhood obesity and metabolic dysfunction in adulthood, both important public health problems in low, medium or high income environments^26^. It is possible that the higher FMI, BMI/age Z-score and FMI of LGA infants reflect a metabolic pattern, which may contribute to the regulation of childhood metabolism and the long-term risk of obesity^26^. In addition, genetic factors, the intrauterine environment and the postnatal environment probably act in combination, having a greater influence than fetal weight gain on the risk of later obesity^27^.

### Strengths and Limitations

The strengths of the present study are the use of population data, the low rate of losses or refusals, and the use of air displacement plethysmography, a useful method for assessing body composition in adolescents^12^, used to assess body fat (FM and FMI) and FFM (FFM and FFMI). And, in order to facilitate comparability with other studies and the communication of our findings, the BMI/age Z-score was also evaluated.

However, it should be considered that air displacement plethysmography is among the limitations of the research, given that it analyzes body density and body composition measurements through the use of equations whose assumptions may not be applicable to all of the cohort participants^28^.

In addition, possible survival bias must be considered. The greatest loss to follow-up occurred among individuals born with or less than 33 weeks of GA, and body composition results may have been underestimated, since the small sample size in this group (n = 37 boys and n = 33 girls) reduced the tests’ statistical power to find associations. About 20% of newborns with or less than 33 weeks of GA in the 2004 cohort died before reaching one year of age^29^. Therefore, body composition results at age 11 could potentially be worse if children born at an earlier GA had survived.

Lastly, other factors can interfere with GA, such as elective cesarean sections and errors in the estimation arising from low-quality or late obstetric ultrasounds, which can overestimate GA in our country by up to 1.8 weeks^30^.

## CONCLUSIONS

Therefore, there was no association between GA and body composition measures at 11 years of age, in either sex. On the other hand, FMI and the BMI/age Z-score were higher in boys born LGA, and FFMI, in girls born LGA, compared to their peers born LGA. However, the long-term consequences of being born LGA are not yet well established in the literature.
