# Supplementary material for: Gestational age, intrauterine growth and body composition at 11 years of age
Source: Rev Saude Publica. 2022 Nov 18;56:116. doi: 10.11606/s1518-8787.2022056004022 (PMC9749740; doi:10.11606/s1518-8787.2022056004022)
Supplement: Translation

# Translation: Idade gestacional, crescimento intrauterino e composição corporal aos 11 anos de idade

**Authors:** Caroline Cardozo Bortolotto, Iná da Silva dos Santos, Juliana dos Santos Vaz, Alicia Matijasevich

**Affiliations:** I Brasil Universidade Federal de Pelotas. Programa de Pós-Graduação em Epidemiologia. Departamento de Medicina Social. Pelotas, RS, Brasil; II Brasil Universidade Federal de Pelotas. Faculdade de Nutrição. Departamento de Nutrição. Pelotas, RS, Brasil; III Brasil Universidade de São Paulo. Faculdade de Medicina. Departamento de Medicina Preventiva. Pelotas, RS, Brasil

**Keywords:** Desenvolvimento Infantil, Composição Corporal, Idade Gestacional, Pletismografia, Estudos de Coortes

## Abstract

**OBJETIVO:**

Avaliar a associação da idade gestacional (IG) e crescimento intrauterino com a composição corporal aos 11 anos de idade.

**MÉTODO:**

Análise de dados da coorte de nascimentos de Pelotas de 2004, cujos desfechos foram massa gorda (MG, kg), índice de massa gorda (IMG, kg/m^2^), massa livre de gordura (MLG, kg), índice de massa livre de gordura (IMLG, kg/m^2^) – medidos por pletismografia por deslocamento de ar –, e índice de massa corporal para idade (IMC/Idade, escore-Z). Sendo as exposições de interesse o índice gestacional (IG) de nascidos com menos de 33 semanas, de 34 a 36 e de 37 a 41, e crescimento intrauterino categorizado em pequeno para a idade gestacional (PIG), adequado (AIG) e grande (GIG). Para comparar médias, utilizou-se análise de variância e, para avaliar a força de associação, regressão linear. As análises foram ajustadas de acordo com variáveis coletadas ao nascer, como renda familiar mensal, características maternas – escolaridade, idade, índice de massa corporal (IMC) pré-gestacional, ganho de peso na gestação, tabagismo na gestação, tipo de parto e paridade – e características dos adolescentes – cor da pele e peso ao nascer. Para análise, o MG e o IMG sofreram transformação logarítmica devido a assimetria dos dados.

**RESULTADOS:**

Foram analisados 3.401 adolescentes, entre meninos e meninas nascidos com menos de 33 semanas, com médias de MG e MLG menores que as dos nascidos(as) a termo. Porém, nas análises ajustadas, não houve associação entre IG e qualquer um dos desfechos em ambos os sexos. Meninos GIG apresentaram IMG 10,5% maior (p = 0,026) e +0,3 escore-Z de IMC/Idade (p = 0,019) em relação aos AIG, e meninas GIG apresentaram +0,3 kg/m^2^ de IMLG (p = 0,039) do que as AIG.

**CONCLUSÃO:**

A IG não se associou à composição corporal aos 11 anos. Entretanto, meninos GIG apresentaram maiores IMG e escore-Z de IMC/Idade e meninas GIG, maior IMLG, quando comparados aos AIG.

## INTRODUÇÃO

A prevalência de excesso de peso em jovens tem aumentado nas últimas décadas. Em 2016, foi estimado que 340 milhões de crianças e adolescentes com idades entre 5 e 19 anos, em todo o mundo, estavam acima do peso ou obesas^1^. No Brasil, inquéritos nacionais têm mostrado, nas últimas três décadas, o aumento na incidência de excesso de peso em crianças e adolescentes entre 6 e 19 anos, independentemente da macrorregião ou renda familiar^2^.

De acordo com a “Teoria da origem fetal das doenças” (*Developmental Origins of Health and Disease* – DoHaD), o excesso de peso e a composição corporal no início da vida estão associados a doenças cardiovasculares e metabólicas na idade adulta^3^. É entre o final da infância e início da adolescência que ocorre uma grande variação na composição corporal^4^, período considerado crítico para o início do desenvolvimento da obesidade em ambos os sexos^5^, embora a distribuição da gordura no corpo ocorra diferentemente entre meninos e meninas^4^. Nisso, além dos fatores relacionados ao estilo de vida, como hábitos alimentares, atividade física e comportamento sedentário, o papel de fatores biológicos, como a idade gestacional (IG), na ocorrência de excesso de peso na infância e adolescência, também tem sido investigado^6-8^.

Estudos longitudinais^7,8^ e transversais^6,9^ mostram que a associação entre IG e composição corporal, avaliada por métodos indiretos – pletismografia por deslocamento de ar^7^ e densitometria de dupla absorção de raio X - Dexa –^6,8,9^, ainda não está bem estabelecida. Enquanto alguns estudos detectaram menores medidas de gordura corporal e massa livre de gordura (MLG) em nascidos a pré-termo – em comparação aos a termo^6,7^–, outros não encontraram associação tanto no início da adolescência, aos 11 anos^9^, quanto no fim dela, aos 18 anos^8^.

Além disso, a relação entre IG, crescimento intrauterino e composição do corpo na adolescência é pouco encontrada na literatura. Dessa forma, o objetivo do presente estudo foi investigar a associação da IG e do crescimento intrauterino com a composição corporal em meninos e meninas de 11 anos de idade, na coorte de nascimentos de Pelotas de 2004.

## MÉTODOS

### Características da Amostra

A pesquisa se desenvolveu com base na análise de dados da coorte de nascimentos de Pelotas de 2004, cidade com 328.275 habitantes e localizada ao sul do Brasil^10^. Nesse ano, foi iniciada uma coorte de nascimentos que incluiu todos os recém-nascidos de partos hospitalares, ocorridos entre primeiro de janeiro e 31 de dezembro, e que acompanhou mães que moravam na área urbana do município e no bairro Jardim América, contíguo a Pelotas mas pertencente ao município vizinho de Capão do Leão. Um total de 4.231 recém-nascidos, ou seja, 99,2% de todos os nascimentos na cidade naquele ano, foram incluídos no estudo, além de entrevistas padronizadas realizadas com as mães durante a estadia no hospital (estudo perinatal) para investigar características socioeconômicas, demográficas e comportamentais. Os recém-nascidos foram examinados nas primeiras 24 horas após o parto pela equipe do estudo, sob a supervisão de um pediatra, sendo acompanhados ao nascer, aos 3, 12, 24 e 48 meses, e aos 6 e 11 anos de idade, com taxas de acompanhamento variando entre 87% e 96% – uma descrição detalhada da metodologia é fornecida em outra publicação^11^.

Para o atual estudo, foram elegíveis aqueles acompanhados aos 11 anos de idade e que tinham informações suficientes para, pelo menos, um dos índices analisados nesta pesquisa; sendo excluídos os gemelares e os que tinham alguma má formação congênita que pudesse interferir na alimentação e deambulação. Considerando isso, aos 11 anos, dos 4.231 membros da coorte, 567 foram perdidos ou recusaram participar do acompanhamento, 84 eram gemelares e 81 não possuíam dados sobre as variáveis essenciais para a realização da pesquisa. Essas perdas ocorreram, por exemplo, por casos em que o adolescente não conseguia ficar em pé para verificação do peso e altura, medidas essenciais para a pletismografia por deslocamento de ar, ou quando não conseguiam ficar dentro da câmera de ar. Somando-se estes casos aos 98 óbitos ocorridos entre o nascimento e os 11 anos de idade, restaram as 3.401 crianças (80,4% do total) incluídas na presente análise.

### Composição Corporal

No acompanhamento feito aos 11 anos, a composição corporal foi avaliada por meio de pletismografia por deslocamento de ar (Bod Pod^®^, Cosmed), realizada por técnicos especificamente treinados; um método seguro, rápido e não invasivo, que pode ser aplicado em diferentes grupos populacionais, como obesos, crianças, adultos e idosos^12^. A fim de avaliá-la, os participantes permaneceram dentro do dispositivo, uma câmara fechada, por alguns segundos, sem se mover. Para, então, medir com precisão adequada e minimizar as disparidades na medição do volume corporal, foi necessário eliminar o efeito do volume de roupas, cabelos, superfície do corpo e pulmões. Em função disso, foram fornecidos aos participantes conjuntos de touca de borracha – como as de natação –, shorts e camisetas de elastano, feitas especialmente para o estudo. Para predição do volume de gás torácico, foi utilizada uma fórmula com base na idade, sexo e altura do participante^13^ e, para estimar a gordura corporal e a MLG, foi usada a equação de Lohman^14^.

Os desfechos deste estudo foram massa gorda (MG) em kg, índice de massa gorda (IMG) em kg/m^2^, MLG em kg, índice de massa livre de gordura (IMLG) em kg/m^2^ e índice de massa corporal (IMC) para idade (em escore-Z). O IMG e IMLG foram calculados a partir da razão, respectivamente, entre a MG (kg) e MLG (kg) e a altura em metros quadrados (m^2^). O IMC/Idade foi calculado pela razão entre o peso em kg e a altura em m^2^. E os escores-Z de IMC/Idade específicos para sexo e idade, calculados de acordo com as curvas de crescimento publicadas pela Organização Mundial de Saúde (OMS) em 2006^15^, usando o software ANTHRO 2007.

O peso foi avaliado por meio da balança de alta precisão (0,01 kg), acoplada ao equipamento BodPod^®^; a altura, foi medida duas vezes, utilizando-se estadiômetro de metal Harpenden (Holtain, Crymych, Reino Unido) com altura máxima de 2,06 m e precisão de 1 mm; obtendo-se enfim a média dessas duas medidas. Nos casos em que a diferença entre as duas aferições foi maior que 1 cm, uma terceira medida era realizada.

### Idade Gestacional e Crescimento Intrauterino

O algoritmo proposto pelo *National Center for Health Statistics* (NCHS)^16^ foi utilizado para estimar a IG ao nascimento, baseada na data da última menstruação (DUM) – sempre que consistente com o peso, comprimento e perímetro cefálico ao nascer, de acordo com as curvas padrão desses parâmetros para cada semana de IG^17^. Caso a estimativa fosse desconhecida ou inconsistente, estimou-se a maturidade clínica baseada no método de Dubowitz^18^_._ Nessa análise, os participantes nascidos antes das 37 semanas de gestação foram classificados como prematuros. Assim, a IG foi categorizada em três grupos: os que nasceram com menos de 33 semanas, que incluem os moderadamente prematuros, muito prematuros e extremamente prematuros; os de 34 a 36 semanas, os prematuros tardios; e os de 37 a 41semanas os recém-nascidos a termo^19^.

O crescimento intrauterino foi definido de acordo com os parâmetros do INTERGROWTH-21^20^, que incluem o peso ao nascer conforme a IG e sexo e a classificação dos participantes em pequenos para a idade gestacional (PIG; peso ao nascer menor que 10%), adequado para a idade gestacional (AIG; peso ao nascer entre 10% e 90%) e grande para a idade gestacional (GIG; peso ao nascer maior que 90%). O peso ao nascer foi coletado usando balanças pediátricas eletrônicas (Harpenden@) com uma precisão de 10g, verificada diariamente pela equipe de pesquisa, sendo considerados de baixo peso ao nascer (BPN) aqueles com menos de 2.500g.

### Potenciais Fatores de Confusão

As variáveis de ajuste foram definidas de acordo com um modelo conceitual criado pelos autores, com base na literatura^21^. Sendo assim, a renda familiar foi obtida pela soma das rendas das pessoas que moravam na mesma casa, no mês anterior à entrevista perinatal, e posteriormente dividida em quintis (Q1, para famílias com menor renda, e Q5, para famílias com maior renda)^22^.

Já as características maternas, obtidas na entrevista perinatal, incluíram a escolaridade, coletada em anos completos com aprovação e classificada em grupos de 0 a 4 anos, de 5 a 8, de 9 a 11 e maior ou igual a 12 anos; idade em anos completos, categorizada em menor de 20 anos, de 20 a 34, e maior ou igual a 35 anos; tabagismo, se fumavam pelo menos um cigarro por dia, todos os dias, em qualquer trimestre da gestação; tipo de parto, se normal ou cesariana, extraído dos registros hospitalares; paridade, ou seja, número de nascimentos vivos ou mortos, categorizada em 0, 1, 2, 3 e maior ou igual a 4; peso no início e final da gestação, extraído da Carteira da Gestante, sendo o último registro considerado o peso final, ou, quando não disponível, o relato da mãe; ganho de peso na gestação, calculado pela diferença entre os pesos final e inicial; IMC pré-gestacional, classificado como baixo peso (≤ 18,49 kg/m^2^), adequado (18,5–24,9 kg/m^2^), sobrepeso (25,0–29,9 kg/m^2^) ou obesidade (≥ 30 kg/m^2^)^23^; e a altura materna, medida no domicílio pela equipe de pesquisa, nos acompanhamentos realizados três meses após o parto.

Por fim, a informação sobre o sexo do adolescente foi coletada direto do prontuário médico de nascimento; e a cor da sua pele, autorreferida pela mãe como branca, parda – ou morena –, preta ou outra, informação obtida nos acompanhamentos feitos aos seis anos de idade. Para as meninas, no acompanhamento dos 11 anos foi perguntado se já haviam tido a menarca. Vale ressaltar ainda que, por apenas 182 mães relatarem cor da pele como “outra”, acabamos agrupando esses adolescentes na categoria “parda”, para fins de análise.

### Análises Estatísticas

As análises estatísticas foram realizadas utilizando-se Stata versão 16.0 (Stata Corp., College Station, EUA). Após descrevida a amostra do acompanhamento aos 11 anos de idade, foram descritos as médias e os intervalos de confiança de 95% (IC95%) de MG, IMG, MLG, IMLG e escore-Z de IMC/Idade, de acordo com a IG e o crescimento intrauterino, separando-se por sexo; para comparar as médias, usou-se a análise de variância (Anova).

Como 251 meninas (15,4%) já haviam tido a menarca, foram realizadas análises para investigar a existência de interação entre a IG e o histórico de menarca sobre os desfechos. Como não houve evidência estatística de interação, todas as meninas foram incluídas nas análises, independentemente do histórico de menarca. Entretanto, houve interação (p < 0,20) entre IG e sexo sobre os desfechos IMLG (p = 0,145) e escore-Z de IMC/Idade (p = 0,092), fazendo com que todas as análises fossem estratificadas por sexo.

A força de associação entre as variáveis independentes e os desfechos foi verificada por regressão linear, através dos coeficientes beta (β) e IC95%; os dados de MG e IMG sofreram transformação logarítmica, por apresentarem distribuição assimétrica; e os coeficientes beta das análises bruta e ajustada são mostrados em sua forma exponencial. Assim, as análises ajustadas foram hierarquizadas, sendo o primeiro nível composto pelas escolaridade e idade maternas e pela renda familiar; o segundo nível, por IMC pré-gestacional, tabagismo na gravidez, ganho de peso na gestação, tipo de parto e paridade; e o terceiro, pela cor da pele do adolescente e peso ao nascer.

A altura atual foi incluída no quarto nível, mas como ela é utilizada na construção do IMG, IMLG e IMC, nas análises para esses desfechos, a altura não foi incluída como um potencial fator de confusão. Da mesma forma, quando a exposição de interesse era o crescimento intrauterino, o peso ao nascer não foi incluído como fator de confusão nos modelos ajustados. Logo, em cada nível, o valor de p foi verificado, sendo removidas as variáveis com o maior valor de p, uma a uma, do modelo de análise. No entanto, foram mantidas na análise, para controle de possíveis efeitos de confusão, as variáveis associadas ao desfecho com valor de p < 0,20. Ao decorrer disso, foi assumido um nível de significância igual a 5% nas análises estatísticas.

### Aspectos Éticos

Todos os acompanhamentos da coorte de nascimentos de Pelotas de 2004 foram aprovados pelo Comitê de Ética em Pesquisa da Faculdade de Medicina da Universidade Federal de Pelotas, afiliado à Comissão Nacional de Ética em Pesquisa (CONEP). Sendo assim, os pais ou responsáveis legais pelas crianças deram consentimento por escrito em cada acompanhamento e foi obtido, também por escrito, o assentimento dos participantes com pelo menos 11 anos de idade (ofício número 889.753).

## RESULTADOS

Na amostra analisada (n = 3.401), 51,7% (n = 1.757) dos participantes eram meninos, sendo cerca de 45% (n = 1.523) nascidos por cesariana, 13,5% (n = 430), pré-termo, e 8,5% (n = 280), PIG (Tabela 1). Em relação às variáveis maternas, a maioria das mães estudaram entre 5 e 8 anos (41,2%) e tinham idades entre 20 e 34 anos (67%). 25,3% delas estavam com sobrepeso e 11,5%, com obesidade. Além disso, a prevalência de tabagismo materno na gestação foi de 26,9%, e ganharam em média 10,5 kg durante a gestação (DP = 5,6).

**Tabela 1:** Características das mães e dos participantes da coorte de nascimentos de Pelotas de 2004, ao nascer, acompanhados aos 11 anos de idade.

Características	Incluídos na análise n = 3.401	

	(n = 3.371)	%
Escolaridade materna (anos completos)^a^		
0–4	497	14,7
5–8	1.387	41,2
9–11	1.162	34,5
≥ 12	325	9,6
Idade materna (anos)	(n = 3.399)	
< 20	648	19,1
20–34	2.279	67,0
≥ 35	472	13,9
IMC pré–gestacional	(n = 2.407)	
Baixo peso (≤ 18.49 kg/m^2^)	91	3,8
Adequado (18,5–24,9 kg/m^2^)	1.432	59,4
Sobrepeso (25,0–29,9 kg/m^2^)	608	25,3
Obesidade (≥ 30 kg/m^2^)	276	11,5
Tabagismo na gestação	(n = 1.757)	
Não	2.487	73,1
Sim	914	26,9
Tipo de parto	(n = 1.878)	
Normal	1.878	55,2
Cesariana	1.523	44,8
Paridade (número de filhos nascidos vivos ou mortos)	(n = 3.400)	
1	1.345	39,6
2	919	27,0
3	539	15,9
≥ 4	597	17,6
Ganho de peso na gestação (kg)	(n = 3.310)	
(média, DP)	10,5 (5,6)	-
Renda familiar (quintis)	(n = 3.401)	
Q1 (menor renda)	657	19,3
Q2	682	20,1
Q3	680	20,0
Q4	730	21,5
Q5 (maior renda)	652	19,2
Cor da pele	(n = 3.384)	
Branca	2.287	67,6
Parda	677	20,0
Preta	420	12,4
Peso ao nascer (g)	(n = 3.401)	
< 2.500 (baixo peso)	260	7,6
2.500–2.999	832	24,5
3.000–3.499	1.369	40,3
3.500–3.999	768	22,6
≥ 4.000	172	5,1
IG (semanas)	(n = 3.186)	
≤ 33	71	2,2
34–36	359	11,3
37–41	2.756	86,5
Crescimento intrauterino	(n = 3.294)	
PIG	280	8,5
AIG	2.446	74,3
GIG	568	17,2

DP: desvio-padrão, IMC: índice de massa corporal, IG: idade gestacional, PIG: pequeno para IG, AIG: adequado para idade gestacional, GIG: grande para idade gestacional.

A Tabela 2 mostra as medianas de MG e IMG e médias de escore-Z de IMC/Idade, com os respectivos IC95%, conforme as exposições de interesse e separadamente para meninos e meninas. Quanto à IG, as menores médias e medianas foram observadas entre os prematuros, em comparação aos a termo. Os meninos nascidos com ou menos de 33 semanas apresentaram medianas de MG e IMG, 6,1 kg e 3,3 kg/m^2^ menores, respectivamente, do que os nascidos a termo (p < 0,001 e p < 0,001). Nas meninas, apenas a MG foi diferente entre as categorias de IG, sendo as medianas das nascidas com ou menos de 33 e com 34 a 36 semanas, 7,6 kg e 9,2 kg menores, também respectivamente, em comparação às nascidas a termo (p = 0,024). O escore-Z de IMC/Idade foi estatisticamente diferente somente entre os meninos, sendo 0,3 ponto menor entre os nascidos com ou menos de 33 semanas, em relação aos nascidos a termo (p < 0,001).

**Tabela 2:** Medidas de tendência central da MG, IMG e IMC para idade (escore-Z), aos 11 anos, conforme o sexo, idade gestacional e crescimento intrauterino, na coorte de nascimentos de Pelotas de 2004.

Variável	Meninos				Meninas			
	
	Total	MG (kg)	IMG (kg/m^2^)	IMC/I (escore-Z)	Total	MG (kg)	IMG (kg/m^2^)	IMC/I (escore-Z)
							
	n	Mediana (IC95%)	Mediana (IC95%)	Mediana (IC95%)	n	Mediana (IC95%)	Mediana (IC95%)	Mediana (IC95%)
IG		**9,6 (3,0 a 26,5)**	**4,5 (1,6 a 11,5)**	**0,8 (0,7 a 0,9)**		**10,0 (3,8 a 27,1)**	**4,6 (1,9ª 11,9)**	**0,7 (0,6 a 0,7)**
(semanas)		**p < 0,001**^**a**^	**p < 0,001**^**a**^	**p < 0,001**^**b**^		**p = 0,024**^**a**^	**p = 0,082**^**a**^	**p = 0,167**^**b**^
≤ 33	38	6,1 (1,9 a 40,3)	3,3 (1,0 a 14,9)	0,3 (- 0,3 a 0,8)	33	7,6 (3,8 a 20,1)	4,0 (1,9 a 9,4)	0,6 (0,2 a 1,0)
34–36	187	7,6 (2,8 a 24,4)	3,7 (1,5 a 10,4)	0,4 (0,2 a 0,7)	172	9,2 (3,0 a 24,8)	4,3 (1,4 a 10,4)	0,5 (0,3 a 0,7)
37–41	1.425	10,1 (3,1 a 26,5)	4,7 (1,6 a 11,5)	0,9 (0,8 a 1,0)	1.331	10,2 (3,9 a 28,1)	4,7 (1,9 a 12,1)	0,7 (0,6 a 0,8)
Crescimento intrauterino		**p = 0,028**^**a**^	**p = 0,021**^**a**^	**p < 0,001**^**b**^		**p = 0,004**^**a**^	**p = 0,006**^**a**^	**p = 0,001**^**b**^
PIG	142	8,1 (2,9 a 21,9)	3,9 (1,6 a 9,5)	0,5 (0,3 a 0,7)	138	8,9 (2,9 a 20,9)	4,1 (1,5 a 9,1)	0,3 (0,1 a 0,5)
AIG	1.286	9,1 (2,9 a 23,7)	4,4 (1,5 a 10,6)	0,7 (0,6 a 0,8)	1.160	9,7 (3,8 a 24,2)	4,5 (1,9 a 10,7)	0,6 (0,5 a 0,7)
GIG	1.704	10,7 (3,1 a 25,3)	5,0 (1,6 a 11,1)	1,0 (0,8 a 1,2)	292	10,7 (3,8 a 25,4)	4,8 (1,9 a 11,1)	0,7 (0,6 a 0,9)

MG: massa gorda; IMG: índice de massa gorda; IMC/I: Índice de Massa Corporal para idade, em escore-Z; IG: idade gestacional; PIG: pequeno para IG; AIG: adequado para IG; GIG: grande para IG; IC95%: Intervalo de confiança de 95%.

^a^ Teste de heterogeneidade não paramétrico Kruskal-Wallis.

^b^ Teste de heterogeneidade paramétrico Anova.

Quanto ao crescimento intrauterino, em ambos os sexos, houve menores médias de escore-Z de IMC/Idade e menores medianas de MG e IMG nos nascidos PIG, em comparação aos AIG. Os meninos PIG apresentaram, em média, menos 0,3 escore-Z de IMC/Idade e menos 8,1 kg e 3,9 kg/m^2^, respectivamente, nas medianas de MG e IMG, em comparação aos AIG. Entre as meninas PIG, a média de escore-Z de IMC foi 0,3 ponto menor e as medianas de MG e IMG, 8,9 kg e 4,1 kg/m^2^ menores, respectivamente, do que entre as nascidas AIG (Tabela 2).

Em relação à MLG, as médias de MLG e IMLG foram menores nos meninos nascidos prematuros em relação aos a termo. Por um lado, os meninos nascidos com ou menos de 33 semanas tiveram, em média, menos 2,2 kg de MLG do que os nascidos a termo, e o IMLG dos meninos nascidos pré-termo foi 0,4 kg/m^2^ menor em relação aos nascidos a termo. Por outro, nas meninas apenas se observou diferenças para MLG, tendo as nascidas com ou menos de 33 semanas, em média, menos 1,2 kg do que as nascidas a termo. Os meninos e meninas nascidos PIG tiveram, respectivamente, menos 0,3 kg e menos 0,6 kg de MLG, em relação aos AIG do mesmo sexo, e o IMLG dos meninos PIG foi 0,2 kg/m^2^menor do que o dos nascidos a termo (Tabela 3).

**Tabela 3:** Medidas de tendência central da MLG e IMLG, aos 11 anos de idade, conforme o sexo, idade gestacional e crescimento intrauterino, na coorte de nascimentos de Pelotas de 2004.

Variável	Meninos			Meninas		
	
	Total	MLG (kg)	IMLG (kg/m^2^)	Total	MLG (kg)	IMLG (kg/m^2^)
					
	n	Média (IC95%)	Média (IC95%)	n	Média (IC95%)	Média (IC95%)
IG		**30,0 (29,8 a 30,2)**	**14,3 (14,2 a 14,3)**		**31,1 (30,8 a 31,4)**	**14,4 (14,3 a 14,4)**
(semanas)		**p < 0,001**^**a**^	**p = 0,001**^**a**^		**p = 0,025**^**a**^	**p = 0,413**^**a**^
≤ 33	38	28,1 (26,0 a 29,5)	13,9 (13,5 a 14,4)	33	30,1 (28,1 a 32,1)	14,6 (13,9 a 15,3)
34–36	187	29,0 (28,3 a 29,7)	13,9 (13,7 a 14,2)	172	30,2 (29,4 a 31,1)	14,2 (14,0 a 14,5)
37–41	1.425	30,3 (30,0 a 30,5)	14,3 (14,2 a 14,4)	1.331	31,3 (30,9 a 31,6)	14,4 (14,3 a 14,5)
Crescimento intrauterino		**p = 0,027**^**a**^	**p = 0,020**^**a**^		**p = 0,018**^**a**^	**p = 0,100**^**a**^
PIG	142	29,4 (28,6 a 30,1)	14,0 (13,8 a 14,2)	138	30,1 (29,1 a 31,1)	14,0 (13,7 a 14,3)
AIG	1.286	29,7 (29,5 a 30,0)	14,2 (14,1 a 14,3)	1.160	30,7 (30,4 a 31,0)	14,3 (14,2 a 14,4)
GIG	1.704	30,6 (30,0 a 31,1)	14,5 (14,3 a 14,6)	292	31,7 (31,0 a 32,4)	14,5 (14,3 a 14,7)

IG: idade gestacional; MLG: massa livre de gordura; IMLG: índice de massa livre de gordura; PIG: pequeno para IG; AIG: adequado para IG; GIG: grande para IG; IC95%: Intervalo de confiança de 95%.

^a^ Teste de heterogeneidade paramétrico: Anova

As Tabelas 4 e 5 apresentam as diferenças entre as médias dos desfechos, de acordo com a IG e o crescimento intrauterino, conforme o sexo. Após ajuste para fatores de confusão, não foi observada associação entre IG e qualquer um dos desfechos, tanto em meninos quanto em meninas (Tabela 4). Em relação ao crescimento intrauterino, meninos nascidos GIG apresentaram IMG 10,5% maior (p = 0,026) e mais 0,3 escore-Z de IMC/Idade (p = 0,019), em relação aos AIG. Nas meninas, houve uma tendência de aumento direto do IMLG com o crescimento intrauterino, tendo as nascidas GIG mais 0,3 kg/m^2^, em comparação às AIG (p = 0,039) (Tabela 5).

**Tabela 4:** Associação entre idade gestacional, composição corporal e escore-Z de índice de massa corporal para idade, aos 11 anos, conforme o sexo, na coorte de nascimentos de Pelotas de 2004.

IG (semanas)	MG (kg)^a^		IMG (kg/m^2^)^a^		IMC/I (escore-Z)		MLG (kg)		IMLG (kg/m^2^)	
				
	β (IC95%)		β (IC95%)		β (IC95%)		β (IC95%)		β (IC95%)	
				
	Bruta	Ajustada	Bruta	Ajustada	Bruta	Ajustada	Bruta	Ajustada	Bruta	Ajustada
Meninos	**p < 0,001**	**p = 0,119**^**b**^	**p < 0,001**	**p = 0,415**^**c**^	**p < 0,001**	**p = 0,206**^**d**^	**p < 0,001**	**p = 0,134**^**e**^	**p < 0,001**	**p = 0,228**^**f**^
≤ 33	-0,4 (-0,6 a -0,1)	0,0 (-0,3 a 0,1)	-0,3 (-0,5 a -0,1)	0,0 (-0,3 a 0,2)	-0,6 (-1,1 - -0,2)	0,0 (-0,6 a 0,5)	-2,2 (-3,8 -0,7)	-0,7 (-1,3 a -0,1)	-0,4 (-0,8 a 0,1)	-0,3 (-0,6 a 0,0)
34–36	-0,2 (-0,3 a -0,1)	0,0 (-0,2 a 0,0)	-0,1 (-0,2 a 0,0)	0,0 (-0,2 a 0,0)	-0,5 (-0,7 a -0,2)	-0,2 (-0,5 a 0,0)	-1,3 (-2,0 -0,6)	0,0 (-1,3 a 1,3)	-0,4 (-0,6 -0,2)	0,0 (-0,6 a 0,6)
37–41	ref.	ref.	ref.	ref.	ref.	ref.	ref.	ref.	ref.	ref.
Meninas	**p = 0,022**	**p = 0,983**^**g**^	**p = 0,070**	**p = 0,714**^**h**^	**p = 0,167**	**p = 0,357**^**i**^	**p = 0,025**	**p = 0,574**^**j**^	**p = 0,831**	**p = 0,206**^**k**^
≤ 33	-0,2 (-0,4 a 0,1)	0,0 (-0,3 a 0,3)	-0,1 (-0,3 a 0,1)	0,0 (-0,3 a 0,3)	-0,1 (-0,6 a 0,4)	0,2 (-0,5 a 0,8)	-1,0 (-2,0 -0,1)	0,3 (-0,5 a 1,0)	0,3 (-0,4 a 0,9)	0,2 (-0,2 a 0,6)
34–36	-0,1 (-0,2 a 0,0)	0,0 (0,1 a 0,1)	-0,1 (-0,2 a 0,0)	0,0 (-0,1 a 0,2)	-0,2 (-0,5 a 0,0)	0,1 (-0,2 a 0,4)	-1,2 (-3,3 - 0,9)	0,1 (-1,6 a 1,7)	-0,2 (-0,4; 0,1)	0,3 (-0,5 a 1,2)
37–41	ref.	ref.	ref.	ref.	ref.	ref.	ref.	ref.	ref.	ref.

ref.: referência

Valores p de tendência linear.

^a^ Foi realizada transformação logarítmica; MG: massa gorda; IMG: índice de massa gorda; MLG: massa livre de gordura; IMLG: índice de massa livre de gordura; IMC: Índice de Massa Corporal para idade, em escore-Z; IC95%: Intervalo de confiança de 95%; ^b^ Ajustado para renda familiar, escolaridade, idade, tabagismo na gestação, ganho de peso na gestação, IMC pré-gestacional e paridade maternos, e peso ao nascer, cor da pele e altura do adolescente. ^c^ Ajustado para renda familiar, escolaridade, idade, tabagismo na gestação, ganho de peso na gestação, IMC pré-gestacional e paridade maternos, e peso ao nascer e cor da pele do adolescente. ^d^ Ajustado para renda, escolaridade, tabagismo na gestação, ganho de peso na gestação, IMC pré-gestacional e paridade maternos e peso ao nascer do adolescente. ^e^ Ajustado para escolaridade, idade, tabagismo na gestação, ganho de peso na gestação, IMC pré-gestacional e paridade maternos, e peso ao nascer, cor da pele e altura do adolescente. ^f^ Ajustado para renda, escolaridade, tabagismo na gestação, ganho de peso na gestação, IMC pré-gestacional, tipo de parto e paridade maternos, e peso ao nascer e cor da pele do adolescente. ^g^ Ajustado para escolaridade, idade, tabagismo na gestação, ganho de peso na gestação, IMC pré-gestacional e paridade maternos, e peso ao nascer e altura do adolescente. ^h^ Ajustado para escolaridade, idade, tabagismo na gestação, ganho de peso na gestação, IMC pré-gestacional e paridade maternos, e peso ao nascer do adolescente. ^i^ Ajustado para escolaridade, tabagismo na gestação, ganho de peso na gestação, IMC pré-gestacional e paridade maternos, e peso ao nascer do adolescente. ^j^ Ajustado para escolaridade, idade da mãe, tabagismo na gestação, ganho de peso na gestação, IMC pré-gestacional e paridade maternos, e peso ao nascer do adolescente. ^k^ Ajustado para tabagismo na gestação, ganho de peso na gestação, IMC materno pré-gestacional e paridade maternos, e peso ao nascer e cor da pele do adolescente.

**Tabela 5:** Associação entre crescimento intrauterino, composição corporal e escore-Z de índice de massa corporal para idade, aos 11 anos, conforme o sexo, na coorte de nascimentos de Pelotas de 2004.

Crescimento intrauterino	MG (kg)^a^		IMG (kg/m^2^)^a^		IMC/I (escore-Z)		MLG (kg)		IMLG (kg/m^2^)	
				
	β (IC95%)		β (IC95%)		β (IC95%)		β (IC95%)		β (IC95%)	
				
	Bruta	Ajustada	Bruta	Ajustada	Bruta	Ajustada	Bruta	Ajustada	Bruta	Ajustada
Meninos	**p = 0,003**	**p = 0,083**^**b**^	**p = 0,003**	**p = 0,026**^**c**^	**p < 0,001**	**p = 0,019**^**d**^	**p = 0,027**	**p = 0,356**^**e**^	**p = 0,020**	**p = 0,193**^**f**^
PIG	-0,1 (-0,2 a 0,0)	-0,1 (-0,2 a 0,0)	-0,1 (-0,2 a 0,0)	-0,1 (-0,2 a 0,0)	-0,2 (-0,4 a 0,0)	-0,2 (-0,4 a 0,1)	-0,4 (-1,2 a 0,4)	-0,3 (-0,9 a 0,3)	-0,2 (-0,4 a 0,1)	-0,2 (-0,5 a 0,1)
AIG	ref.	ref.	ref.	ref.	ref.	ref.	ref.	ref.	ref.	ref.
GIG	0,1 (0,0 a 0,2)	0,1 (0,0 a 0,2)	0,1 (0,0 a 0,2)	0,1 (0,1 a 0,2)	0,3 (0,1 a 0,5)	0,3 (0,1 a 0,5)	0,8 (0,2 a 1,4)	0,3 (-0,2 a 0,8)	0,3 (0,1 a 0,5)	0,2 (0,0 a 0,4)
Meninas	**p = 0,005**	**p = 0,178**^**g**^	**p = 0,006**	**p = 0,067**^**h**^	**p = 0,001**	**p = 0,050**^**i**^	**p = 0,018**	**p = 0,088**^**j**^	**p = 0,100**	**p = 0,039**^**k**^
PIG	-0,1 (-0,2 a 0,0)	-0,1 (-0,2 a 0,0)	-0,1 (-0,2 a 0,0)	-0,1 (-0,2 a 0,0)	-0,3 (-0,5 a 0,0)	0,0 (0,0 a 0,2)	-0,6 (-1,6 a 0,5)	-0,3 (-1,0 a 0,5)	-0,3 (-0,6 a 0,0)	-0,1 (-0,5 a 0,2)
AIG	ref.	ref.	ref.	ref.	ref.	ref.	ref.	ref.	ref.	ref.
GIG	0,0 (0,0 a 0,2)	0,0 (0,0 a 0,2)	0,0 (0,0 a 0,1)	0,1 (0,0 a 0,2)	0,2 (0,0 a 0,3)	0,0 (0,0 a 0,0)	1,1 (0,3 a 1,8)	0,6 (0,0 a 1,1)	0,2 (0,0 a 0,5)	0,3 (0,1 a 0,6)

ref.: referência. Valores p de tendência linear. ^a^ Foi realizada transformação logarítmica; MG: massa gorda; IMG: índice de massa gorda; MLG: massa livre de gordura; IMLG: índice de massa livre de gordura; IMC: Índice de Massa Corporal para idade, em escore-Z; IG: idade gestacional; PIG: pequeno para IG; AIG: adequado para IG; GIG: grande para IG; IC95%: Intervalo de confiança de 95%; ^b^ Ajustado para renda familiar, escolaridade, idade, tabagismo na gestação, ganho de peso na gestação, IMC pré-gestacional e paridade maternos, e cor da pele e altura do adolescente. ^c^ Ajustado para renda familiar, escolaridade, idade, tabagismo na gestação, ganho de peso na gestação, IMC pré-gestacional e paridade maternos, e cor da pele do adolescente. ^d^ Ajustado para renda familiar, escolaridade, tabagismo na gestação, ganho de peso na gestação, IMC pré-gestacional e paridade maternos. ^e^ Ajustado para renda familiar, escolaridade, tabagismo na gestação, ganho de peso na gestação, IMC pré-gestacional e paridade maternos, e peso ao nascer, corda pele e altura do adolescente. ^f^ Ajustado para renda familiar, escolaridade, tabagismo na gestação, ganho de peso na gestação, IMC pré-gestacional e tipo de parto maternos, e peso ao nascer e cor da pele do adolescente. ^g^ Ajustado para escolaridade, idade, tabagismo na gestação, ganho de peso na gestação, IMC pré-gestacional e paridade maternos, e altura do adolescente. ^h^ Ajustado para escolaridade, idade, tabagismo na gestação, ganho de peso na gestação, IMC pré-gestacional, tipo de parto e paridade maternos, e cor da pele do adolescente. ^i^ Ajustado para escolaridade, tabagismo na gestação, ganho de peso na gestação, IMC pré-gestacional e paridade maternos. ^j^ Ajustado para escolaridade, idade, IMC pré-gestacional e paridade maternos, e peso ao nascer, cor de pele e altura do adolescente. ^k^ Ajustado para tabagismo na gestação, IMC pré-gestacional e paridade maternos, e peso ao nascer e cor da pele do adolescente.

## DISCUSSÃO

Este estudo analisou a associação da IG e do crescimento intrauterino com a composição corporal no início da adolescência, ou seja, aos 11 anos de idade. Após ajustes para fatores de confusão, apenas o IMG e escore-Z de IMC/Idade foram maiores entre meninos nascidos GIG, em comparação aos AIG; e, entre as meninas, apenas o IMLG das nascidas GIG foi mais elevado do que o das AIG.

A não associação verificada da IG com os desfechos é consistente com os achados de outros autores^8,9^. Na coorte de nascimentos de Pelotas de 1993, por exemplo, não foi observada associação entre IG e MG, MLG – mensurados pela pletismografia de deslocamento de ar – ou IMC, em ambos os sexos ao final da adolescência, aos 18 anos de idade^8^. Da mesma forma, um estudo de caso-controle, realizado na Espanha, não encontrou diferença nas MG e MLG entre os nascidos pré-termo e a termo de sete a 11 anos, grupo controle avaliado pelo Dexa^9^.

Em contrapartida, na mesma coorte, foram verificados menores valores de MG, IMG, MLG e IMLG, medidos por pletismografia por deslocamento de ar, nos meninos de seis anos de idade nascidos entre 34 e 36 semanas, isso em comparação aos a termo^8^. Outros estudos, com participantes de 8 a 12 anos de idade, no Reino Unido^6^, e de 11 anos, na Inglaterra^7^, ambos realizados pelo Dexa, também identificaram menores conteúdos de gordura corporal entre adolescentes nascidos pré-termo^6,7^.

A plausibilidade da associação entre nascimento pré-termo e menor gordura corporal na infância e adolescência baseia-se no fato de que o armazenamento de energia – gordura e glicogênio –, de nutrientes, além da maior deposição de MG e MLG^24^, ocorrem principalmente no último trimestre da gestação. Desse modo, a interrupção da gestação antes das 37 semanas levaria a uma baixa reserva de energia e nutrientes nos nascidos prematuros^3^.

Os mecanismos responsáveis pela diferença na composição do corpo de crianças nascidas prematuras são provavelmente multifatoriais, como fatores que influenciam o manuseio ou a disponibilidade de nutrientes, doenças concomitantes, como infecção e doenças pulmonares, e influências hormonais, por exemplo, o uso de corticosteroides pós-natais^24^. Logo, como sugerido por Fewtrell et al.^6^, é possível que a interrupção prematura da deposição de gordura intraútero nos nascidos pré-termo tenha efeito de longo prazo no ganho subsequente de gordura corporal.

Embora a evidência sobre a associação entre IG e composição do corpo na adolescência ainda seja escassa, estudos mostram menor MG entre os pré-termo na infância^6,8^ e uma inversão do efeito da IG sobre a MG na idade adulta^8^, de modo que os nascidos prematuros apresentam mais MG em relação aos nascidos a termo quando adultos. Essa inversão de efeito ao longo da vida pode estar relacionada a mecanismos mediadores, como o nível de escolaridade, o consumo de álcool, atividade física e hábitos alimentares^25^.

As interações entre IG e sexo para o IMLG e escore-Z de IMC/Idade, observadas em nosso estudo, possivelmente decorrem do fato de que a maturação sexual ocorre de forma diferente entre meninos e meninas^4^. Os fenômenos puberais apresentam variabilidade quanto às idades de início e término e à velocidade e magnitude com que se expressam, o que pode influenciar as mudanças antropométricas e de composição corporal que se diferenciam no processo de crescimento e desenvolvimento da adolescência^4^. Nesse período, em ambos os sexos ocorre significativo aumento da MG, mas, nos meninos, ele é mais lento, sendo superado pelo ganho em MLG^4^.

Em relação ao crescimento intrauterino, nascer GIG está associado à obesidade infantil e disfunção metabólica na idade adulta, ambos importantes problemas de saúde pública, em ambientes de baixa, média ou alta renda^26^. É possível que os maiores IMG, escore-Z de IMC/Idade e IMLG dos nascidos GIG reflitam um padrão metabólico, podendo contribuir para a regulação do metabolismo na infância e o risco de obesidade a longo prazo^26^. Além disso, fatores genéticos, o ambiente intraútero e o ambiente pós-natal provavelmente atuam em combinação, tendo maior influência do que o ganho de peso fetal, no risco de obesidade posterior^27^.

### Pontos Fortes e Limitações

Os pontos fortes do presente estudo estão no uso de dados populacionais, na baixa taxa de perdas ou recusas e no uso de pletismografia por deslocamento de ar, um método proveitoso para a avaliação da composição corporal em adolescentes^12^, utilizado na avaliação da gordura corporal (MG e IMG) e da MLG (MLG e IMLG). E, visando facilitar a comparabilidade com outros estudos e a comunicação de nossos achados, também foi avaliado o escore-Z de IMC/Idade.

Contudo, deve-se considerar que a pletismografia por deslocamento de ar está entre as limitações da pesquisa, haja vista que ela analisa a densidade corporal e as medidas de composição corporal por meio do uso de equações cujos pressupostos podem não ser aplicáveis a todos os participantes da coorte^28^.

Além disso, o possível viés de sobrevivência deve ser considerado. A maior perda de acompanhamento ocorreu entre os indivíduos nascidos com ou menos de 33 semanas de IG, podendo estar subestimados os resultados de composição corporal, visto que o pequeno tamanho amostral nesse grupo (n = 37 meninos e n = 33 meninas) reduziu o poder estatístico dos testes para encontrar associações. Cerca de 20% dos recém-nascidos com ou menos de 33 semanas de IG da coorte de 2004 morreram antes de completar um ano de idade^29^. Logo, resultados da composição corporal aos 11 anos poderiam ser potencialmente piores, se crianças nascidas em IG mais precoces tivessem sobrevivido.

Em último lugar, outros fatores podem interferir na IG, como cesarianas eletivas e erros na estimativa oriunda de ultrassons obstétricos de baixa qualidade ou realizados tardiamente, os quais podem superestimar a IG em nosso meio em até 1,8 semana^30^.

## CONCLUSÕES

Portanto, não houve associação entre a IG e as medidas de composição corporal aos 11 anos, em ambos os sexos. Já o IMG e o escore-Z de IMC/Idade foram maiores nos meninos nascidos GIG, e o IMLG, nas meninas nascidas GIG, em comparação a seus pares nascidos AIG. Contudo, as consequências a longo prazo por se nascer GIG ainda não estão bem estabelecidas na literatura.
